# Does Community-Level Social Capital Predict Decline in Instrumental Activities of Daily Living? A JAGES Prospective Cohort Study

**DOI:** 10.3390/ijerph16050828

**Published:** 2019-03-07

**Authors:** Satoko Fujihara, Taishi Tsuji, Yasuhiro Miyaguni, Jun Aida, Masashige Saito, Shihoko Koyama, Katsunori Kondo

**Affiliations:** 1 Department of Public Health, Graduate School of Medicine, Chiba University, Chuo-ku, Chiba 260-8670, Japan; kkondo@chiba-u.jp; 2 Center for Preventive Medical Sciences, Chiba University, Chuo-ku, Chiba 260-8670, Japan; tsuji.t@chiba-u.jp; 3 Department of Gerontological Evaluation, Center for Gerontology and Social Science, National Center for Geriatrics and Gerontology, Obu, Aichi 474-8511, Japan; y.miyaguni@ncgg.go.jp; 4 Department of International and Community Oral Health, Tohoku University Graduate School of Dentistry, Aoba-ku, Sendai, Miyagi 980-8575, Japan; j-aida@umin.ac.jp; 5 Department of Social Welfare, Nihon Fukushi University, Mihama-cho, Chita-gun, Aichi 470-3295, Japan; masa-s@n-fukushi.ac.jp; 6 Department of Cancer Epidemiology, Cancer Control Center, Osaka International Cancer Institute, Chuo-ku, Osaka 541-8567, Japan; shihoko167362@gmail.com

**Keywords:** physical function, civic participation, multilevel analysis

## Abstract

Instrumental activities of daily living (IADL) represent the most relevant action capacity in older people with regard to independent living. Previous studies have reported that there are geographical disparities in IADL decline. This study examined the associations between each element of community-level social capital (SC) and IADL disability. This prospective cohort study conducted between 2010 and 2013 by the Japan Gerontological Evaluation Study (JAGES) surveyed 30,587 people aged 65 years or older without long-term care requirements in 380 communities throughout Japan. Multilevel logistic-regression analyses were used to determine whether association exists between community-level SC (i.e., civic participation, social cohesion, and reciprocity) and IADL disability, with adjustment for individual-level SC and covariates such as demographic variables, socioeconomic status, health status, and behavior. At three-year follow-up, 2886 respondents (9.4%) had suffered IADL disability. Residents in a community with higher civic participation showed significantly lower IADL disability (odds ratio: 0.90 per 1 standard deviation increase in civic participation score, 95% confidence interval: 0.84–0.96) after adjustment for covariates. Two other community-level SC elements showed no significant associations with IADL disability. Our findings suggest that community-based interventions to promote community-level civic participation could help prevent or reduce IADL disability in older people.

## 1. Introduction

Instrumental activities of daily living (IADL) represent the most relevant action capacity in older people for independent living [1,2,3,4]. IADL decline is a predictor of cognitive ability decline [5] and of institutionalization [6,7], and these lead to higher health care costs [8,9]. Previous studies have reported that there are geographical disparities in IADL decline [10,11]. It is possible that these substantial geographical disparities are affected by the contribution of community-level factors, but the cause of the disparity is unknown.

Social capital has recently attracted attention as a factor forming part of a community environment, and is considered to contribute to health inequality among regions [12,13,14]. Social capital has been defined as “resources that are accessed by individuals as a result of their membership of a network or a group” in the field of public health [15]. Social capital has been defined in two ways [15]. There is individual-level social capital, which includes resources derivable from the social networks of an individual; there is also group-level social capital, which pertains to the social network as a whole. Previous studies reported that community-level social capital is protective of individual-level health outcomes [16,17,18,19,20,21,22,23]. Health studies take account of community-level social capital because community-level social capital may affect everyone in the community, bringing disparities in health outcomes between communities [16,24].

Several pathways through which social capital exerts the following contextual effects on individual health link community-level social capital to individual health outcomes: (i) Social contagion, the notion that behaviors spread more quickly through a tightly knit social network; (ii) informal social control, the ability of adults in a community to maintain social order; and (iii) collective efficacy, the group-level analog of self-efficacy, or the collective ability to mobilize and take collective action [15]. Regarding the behavioral pathway, several studies reported that social capital is considered to affect health behaviors through social contagion and collective efficacy [16,17,18,25,26].

Previous studies on the relationship between the three elements (i.e., civic participation, social cohesion, and reciprocity) of community-level social capital and health have suggested that civic participation is protective, while the other two elements are not protective [17,23]. Therefore, it is possible that these elements of the community-level social capital are differently related to health. In terms of the relationship between community-level social capital and IADL disability, it was hypothesized that older people living in locations with higher community-level civic participation can easily obtain health information through social contagion, which may lead to better maintenance of their IADL. However, the association of community-level social capital with IADL decline and its regional disparities remains unknown even though it is certain that social capital affects health outcomes.

This study proposed to use multilevel survival analysis to examine the prospective associations between each element of community-level social capital and the incidence of IADL disability in older people in various municipalities throughout Japan. It is possible that community-based interventions to promote community-level social capital could help prevent IADL disability or reduce its incidence. The outcomes obtained from the present investigation will add to the body of knowledge pertaining to the prevention and reduction of regional disparities with regard to IADL decline in older people.

## 2. Materials and Methods

### 2.1. Data Sources and Participants

The data were obtained from the Japan Gerontological Evaluation Study (JAGES). This project investigated the social determinants of health among non-institutionalized and functionally independent people aged ≥65 years [27]. The sample investigated by the JAGES included only participants who are not eligible to receive public long-term-care insurance benefits. This study used panel data from two longitudinal surveys. The baseline survey was conducted between August 2010 and January 2012. A total of 141,452 community-dwelling people aged 65 years and older were randomly selected from 24 municipalities in nine prefectures in Japan and mailed a self-administered questionnaire. A total of 92,272 people responded (response rate, 65.2%). To ensure all relevant community-level social-capital variables were assessed, we excluded 7163 responses for the following reasons: (i) Missing information on sex, age, or address; and (ii) having physical or cognitive disabilities. Moreover, owing to a lack of information regarding community areas and to avoid non-precise values due to small samples, we excluded 149 community areas with <50 respondents each (a total of 5476 responses). A total of 25,509 responses were excluded owing to a lack of information regarding individual-level social capital. Thus, the final sample consisted of 54,124 valid responses from people living in 381 communities whose boundaries were primarily based on the demarcations of school districts. We aggregated individual responses into school districts to assess social capital at the community-level. School districts were selected as the unit of community in this paper for two reasons [23]. First, in most regions, a school district represents the size of territory that older people can easily traverse on foot or by bicycle, and community organizations, such as senior citizens clubs and sports clubs, conduct their activities within each school district. Thus, using school districts as the sampling unit, we evaluated regional variability in social capital within each municipality, which may guide local public-health practitioners in their work. Second, the school district is the smallest-size area for which it was possible to maintain sufficient precision in the aggregated information, regarding the number of samples within each community.

**Figure 1:**
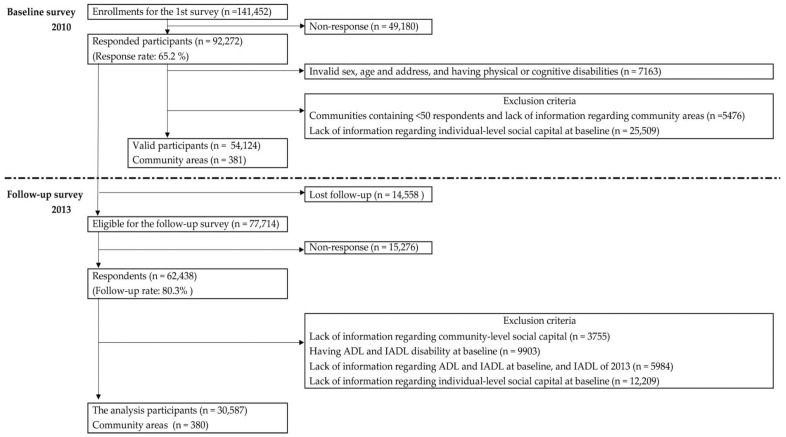
Flow chart showing participation in surveys of 2010 and 2013.

The follow-up survey was conducted between October 2013 and December 2013. Out of the 77,714 baseline respondents, 14,558 people were excluded at follow-up because they no longer met the requirements for the subject of the study (for example, an excluded respondent may live in a municipality where we were unable to receive cooperation from the local government), and were either deceased, or had relocated, or required long-term care. The self-administered questionnaires used for the follow-up survey were subsequently mailed to the same respondents as previously. In total, 62,438 people completed both the 2010 and 2013 questionnaires. Out of the 62,438 respondents, 3755 people were excluded because information on social capital could not be followed up, and 9903 were excluded owing to activities of daily living (ADL) and IADL limitations at baseline; namely, having scored below 4 on the 5-item Tokyo Metropolitan Institute of Gerontology Index of Competence (TMIG-IC) [3]. Furthermore, we excluded 5984 people because information regarding their ADL and IADL scores from 2010 and/or their IADL scores in 2013 were missing. In total, 12,209 responses were excluded owing to a lack of information regarding individual-level social capital at baseline. After all exclusions, 30,587 respondents from 380 communities were included in our analyses (Figure 1).

The JAGES respondents were advised that participation in the study was voluntary and that completing and returning the self-administered questionnaire indicated consent to participation in the study. Ethics approval was obtained from the Ethics Committee at Nihon Fukushi University, Japan (No. 10-05).

### 2.2. Dependent Variable: IADL

IADL was measured using the five-item TMIG-IC [3], which is based on the Lawton IADL scale [28]. It examines five activities that people may perform in daily life, such as (i) using public transportation, (ii) shopping for daily necessities, (iii) preparing meals, (iv) paying bills, and (v) managing deposits at a bank or post office. Each item was scored 1 for yes (able to do) or 0 for no (unable to do). Subjects whose total score was ≤4 were defined as dependent, and those with a total score of 5 as independent [29].

#### 2.2.1. Main Predictor Variable: Community-Level Social Capital

We assumed that respondents living in the same communities were all exposed to the same degree of community-level social capital, whose contextual effect was determined using variables defined in a previous study [23], applied as follows. The variables constituting community-level social capital were obtained through factor analysis [17,23]. First, the rates of each individual-level social-capital response in each small district were calculated. Then, taking each of the 381 communities as the units of analysis, factor analyses was conducted, and three factors were obtained, namely, civic participation (i.e., participation in a volunteer group, a sports group, and a hobby activity), social cohesion (i.e., community trust and attachment), and reciprocity (i.e., receiving/providing emotional support or receiving instrumental support). The factor scores for each community were used as variables for community-level social capital.

#### 2.2.2. Predictor Variable: Individual-Level Social Capital

In 2010, we assessed individual-level social capital as participation in civic life, social cohesion, and reciprocity. Civic participation was assessed using the following question: “How often do you participate in a volunteer group, a sports group, or a hobby activity?” The responses were categorized into “yes” or “no” based on the participation frequency using of the following options: (1) Almost every day, (2) twice or thrice a week, (3) once a week, (4) once or twice a month, (5) a few times a year, and (6) never. The response was categorized as “yes” if the participants participated in any of the three groups at least once a month or more and “no” if there were a few times a year participation or never. Social cohesion was assessed using the following questions: “Do you trust the people who live in your local area?” “Do you think that it is important to be helpful to other people in your local area?” and “Do you have an attachment to your local area?” [17,23]. The response categories for social cohesion variable were a yes answer to at least one of these three questions and a no to all. Reciprocity was assessed using the following questions: “Do you have someone who listens to your concerns and complaints?” (examining the receipt of emotional support), “Do you listen to someone else’s concerns and complaints?” (providing emotional support), and “Do you have someone who looks after you when you are sick?” (receiving instrumental support). The response categories used for reciprocity were a yes to at least one of the three questions and no to all.

### 2.3. Covariates

Potential confounding factors that were considered included demographic variables, socioeconomic status, and health status and behavior. In the 2010 survey, these were included in the analyses as covariates. The demographic variables and socioeconomic status indicators included sex, age (65–69, 70–74, 75–79, 80–84, or ≥ 85 years), marital status (married, widowed, divorced, or never married), educational attainment (<10, 10–12, or ≥ 13 years), annual household income (<2,000,000, 2,000,000–3,999,999, or ≥4,000,000 yen (Japanese Yen); 1 million yen is equivalent to 10,000 US$). The measure of health status included the presence of illnesses and depression symptoms, which were assessed using a validated cut-off (5 or more) with the Geriatric Depression Scale (GDS) [30]. Health behavioral information included body mass index (<18.5, 18.5–24.9, or ≥25.0 kg/m^2^), smoking habit, alcohol consumption, daily walking time, and frequency of going outside. Community-level covariates include the community-level average annual household income, and the population density per km^2^ of inhabitable area for all community areas, categorized into quartile categories (<2040, 2040–6852, 6853–10,122, and ≥10,123 persons per km^2^) and area, which was also classified into quartiles (<1.256, 1.256–2.2563, 2.2564–5.134, and ≥5.135 km^2^).

### 2.4. Statistical Analysis

We used multilevel logistic-regression analyses to determine associations between community-level social capital and the incidence of IADL disability. The fixed parameters of the individual and community were converted into odds ratios (OR) with 95% confidence intervals (CI). To determine the contextual effects of community-level social capital variables on the incidence of IADL disability, an adjustment of the compositional individual factors was needed. Three models of analysis were used, as follows: First, each community-level (contextual) social capital variable was incorporated into the model without adjustment (Model 1). Second, community-level average annual household income, population density, area, individual-level social capital, sex, age, marital status, educational attainment, and annual household income were added (Model 2). In the final model (Model 3), the presence of illnesses, depression symptoms, body mass index, smoking habit, alcohol consumption, daily walking time, and frequency of going outside were added. If data were missing for any explanatory variable, the corresponding observation was assigned to the category of the missing variable.

Next, to confirm the robustness of our finding, we performed a sensitivity analysis, treating the community-level social capital variables as categorical by the quartile; subgroup analyses were stratified by sex and age (65–74 or ≥75 years old). The significance level was set at *p* < 0.05. We used SPSS V.23.0 (IBM Corp, Armonk, New York, USA) for factor analysis and STATA V.14 (Stata Corp, College Station, TX, USA) for multilevel analysis.

## 3. Results

Table 1 shows descriptive statistics for the individual variables. Of 30,587 respondents included in the analyses, 13,919 were men and 16,668 were women; the average age was 71.8 years (standard deviation [SD] = 5.1). Of the total number of respondents, 2886 (9.4%) reported incidence of IADL disability. Participants who were men, were older, had less education, were depressed, were smokers, walked more rarely, and went outside less frequently had a higher incidence of IADL disability.

Exploratory factor analysis (Table 2) suggested that three factors (eigenvalues: 3.10, 1.97, and 1.25) are composed of the nine variables; these factors had a cumulative contribution of 57.7%. The first of these factors was largely associated with community trust, norms of reciprocity and community attachment (α = 0.84); it was termed social cohesion. The second factor was strongly associated with participation in sports groups, volunteer groups, and hobby activities (α = 0.72). This factor was termed civic participation. The third factor was strongly associated with receiving/providing emotional support and receiving instrumental support (α = 0.72) and was termed reciprocity.

Table 3 shows descriptive statistics for the community-level variables. When the proportions of the incidence of IADL disability (mean ± SD, 8.0 ± 4.9) were calculated for each community area, the range was 0–30%. When the factor score (mean factor score ± SD, −0.0021 ± 0.93 in social cohesion, −0.0027 ± 0.92 in civic participation, and −0.0014 ± 0.91 in reciprocity) of each community-level social capital was calculated for each community area, the ranges were −2.67–2.61, −2.97–3.87, and −3.79–2.05, respectively.

Table 4 shows the results of multilevel logistic-regression analyses. The null model, which had no predictors, showed significant variation in the incidence of IADL disability between communities (*σ*^2^ = 0.26). The crude model (Model 1) found that all community-level social capital variables were statistically associated with the incidence of IADL disability. The OR for this result was 0.81 (95% CI: 0.76–0.85) per 1 SD increase in civic participation score. The ORs for social cohesion and reciprocity were 1.15 (95% CI: 1.07–1.24) and 1.10 (95% CI: 1.00–1.19), respectively. After adjusting for community-level average annual household income, population density, area, individual-level social capital, sex, age, and socioeconomic status (Model 2), only civic participation showed a significant association with the incidence of IADL disability (OR: 0.89, 95% CI: 0.83–0.95). In the full model (Model 3), a similar tendency was observed with regard to the association between community-level social capital and the incidence of IADL disability (OR: 0.90, 95% CI: 0.84–0.96).

The results of the sensitivity analysis are shown in Table S1 (see supplementary material). The results of the analyses categorizing the community-level social capital variables by the quartile were similar to those when the community-level social capital was the continuous variable. Subgroup analyses results are presented in Table S2. There was no significant association in men, but a similar relationship was found in women and all age groups. 

## 4. Discussion

To the best of our knowledge, this study is the first to use a multilevel analysis to explore the associations between each element of community-level social capital and the incidence of IADL disability in older people residing in multiple municipalities. Our results suggest that living in communities with higher community-level civic participation was associated with lower incidence of IADL disability, even after adjustment was conducted for covariates, including variables of individual-level social capital. In addition, the results of the sensitivity and subgroup analyses imply that the robustness of the finding for the association between community-level civic participation and IADL disability.

Each increase of standard deviation in community-level civic participants reduced the incidence of IADL disability by 10%. The OR for community-level civic participation had a reciprocal of 1.11 (1/0.90). It is worth noting that the mitigation relationship of 1 SD increases in community-level civic participation, estimated by the reciprocals of the OR (1.11), was comparable with the adverse effect of the presence of illnesses (OR = 1.13).

The results of the present study bear some similarities to those of previous longitudinal studies [31,32]. Such studies have suggested that higher individual-level civic participation, which is an aspect of social capital, lowers the incidence of IADL disability. However, these previous studies focused only on individual-level civic participation. Community-level social capital is also important for supporting older people in their maintenance of health and well-being, as they may spend much of their time in their community [23]. Therefore, in the examination of associations between social capital and health, it is important to examine not only individual-level but also community-level social capital. The present study adds evidence that supports the association between community-level civic participation and the incidence of IADL disability, suggesting that increases in community-level social capital prevents or reduces the incidence of IADL disability. This may lead to interventions to increase community-level civic participation as early intervention and population strategy for the prevention of the decline of cognitive abilities and institutionalization.

Social capital has disadvantages despite the health benefits associated with it [33,34]. Portes has demonstrated four downsides of social capital: The exclusion of outsiders, excess claims made on group members, restrictions on individual freedoms, and a downward-leveling of norms [33]. A previous study conducted in Japan suggested that too strong a level of social cohesion can result in the social exclusion of those who are deemed to be “outsiders”, which increases depressive symptoms for residents whose hometowns of origin differ from the communities in which they currently reside [35]. We suspect that too strong a level of social cohesion can result in the social exclusion of those who are deemed to be outsiders, and that this eventuality can lead to negative physical and mental impact and may not protect IADL. In this study, reciprocity might have been incapable of eliminating the reverse causality of increased support by people with lower IADL. Long-term studies must be conducted to identify the possible reasons for these results.

There are several pathways linking social capital to health outcomes through which social capital exerts the following contextual effects on individual health: Social contagion, informal social control, and collective efficacy [15]. Regarding these pathways, in the present study, social contagion, in particular, might explain the association between community-level civic participation and decline of IADL. It is presumed that there are many people interested in health in regions where civic participation is active. Social contagion may make it easier for people who do not participate in civic life to have useful information on health behaviors and, in turn, prevent the decline of IADL. In addition, it has been reported that through civic participation, people facilitate an increase of other social activities, which leads to the spread of such activities in spreading social activities in communities [36]; those who do not participate in the given social activity also benefit, and these effects can lead to increasing the health of a community. Moreover, via collective efficacy, in communities of higher social capital, residents are connected to each other, and their health facilities, systems, and service are more substantial [15], which may be what leads to the prevention or reduction of the incidence of IADL disability. In this study, the incidence of IADL disability was found to be low in areas with higher population density. Many services are readily available and transportation is relatively well developed in localities with higher population densities. This attribute could lead to increased opportunities of stepping out of the home environment and could prevent or reduce IADL decline. For the above reasons, social contagion and collective efficacy are the important pathways that link community-level civic participation to the prevention or reduction of IADL decline. On the other hand, social contagion and collective efficacy may not pertain to the pathways that link community-level social cohesion and reciprocity to the prevention or reduction of IADL decline. This might explain the non-significant association of community-level social cohesion and community-level reciprocity with the IADL decline.

Previous studies suggested that at the individual level, higher socioeconomic status is protective of health problems among older adults [37,38]. However, in this study, higher income communities were associated with higher IADL disability. After the community-level average annual household income was added to the crude model, the IADL decline was found to be progressively lower in correspondence to increasing community-level average annual household incomes. Conversely, after additional variables were adjusted for, the result was reversed. One of the variables may have been confounded, but the details remain unclear; hence, further studies should be conducted to identify the possible reasons for these results.

The present study has certain noteworthy strengths. The size of the sample is an important point, as it enabled the consideration of a wide range of communities and their contextual characteristics, namely, 380 communities. Thus, the present study was able to account for the effects of community factors in a more thorough way. In addition, the survey had a prospective cohort design and used panel data. This design was suitable for inferring causality, to a greater degree than the design of previous cross-sectional studies.

However, this study also has several limitations. First, the possibility of response bias must be noted because all measures were assessed with a self-administered questionnaire, resulting in possible biases such as social desirability [39], which may have decreased the prevalence of lower social capital in certain areas, as calculated from the responses to the questionnaire, and therefore to certain inaccuracies. Non-respondents tended to present a higher prevalence of disability [40]. Since this study was conducted via mail survey, there may have been people with disabilities among the non-respondents. Therefore, a greater inclusion in the analysis of regions with lower social capital and higher prevalence of disabilities could narrow confidence intervals. Second, it might be suspected that the study sample was made up of a relatively healthy group, because it was limited to those who responded to both the baseline and follow-up surveys. Therefore, although the results appeared to contain an underestimation of the association between community-level civic participation and the incidence of IADL disability, the significance was confirmed nevertheless. Third, relative to decline of physical functions, IADL covers the items in the basic activities of daily living. Further study should involve long-term follow-up of a population, addressing outcomes such as dementia and long-term care. Finally, although, it has been suggested and confirmed reproducibility of a positive influence on the association between higher social capital and various health indicators using multilevel analysis [16,17,18,19,20,21,22,23], community-level social capital might be a proxy for the community-level variables, which were not examined in this study, besides population density and community-level average annual home income. However, it has been reported that social capital was effective in preventing the onset of disability and cognitive decline in community-based intervention study [41,42]. Therefore, even if social capital is in part a proxy for other factors, there is a high possibility that community social capital promotes health.

## 5. Conclusions

This cohort study of older Japanese people found that living in a community area which has a higher community-level civic participation is associated with a lower incidence of IADL disability than living in a community area with lower community-level civic participation, after adjusting for individual-level social capital and other covariates. Community-based interventions to promote civic participation may help prevent or reduce the incidence of IADL disability in older people.

## Figures and Tables

**Table 1:** Baseline characteristics of respondents and newly decline of IADL (*n* = 30,587).

Variables	Newly Decline of IADL				Total
	No		Yes		
Individual-Level Variable	Number	%	Number	%	Number
Sex					
Men	12,034	86.5	1885	13.5	13,919
Women	15,667	94.0	1001	6.0	16,668
Age (years)					
65–69	11,628	93.1	858	6.9	12,486
70–74	8885	91.7	809	8.4	9694
75–79	5029	88.7	643	11.3	5672
80–84	1781	81.1	416	18.9	2197
≥85	378	70.3	160	29.7	538
Marital status					
Married	20,529	90.1	2264	9.9	22,793
Windowed	5039	91.2	485	8.8	5524
Divorced	960	96.4	36	3.6	996
Never married	602	95.3	30	4.8	632
Educational attainment (years)					
<10	10,169	87.9	1400	12.1	11,569
10–12	10,969	92.5	895	7.5	11,864
≥13	6002	92.3	501	7.7	6503
Annual household income (yen)					
<2,000,000	10,724	90.1	1182	9.9	11,906
2,000,000–3,999,999	10,861	91.3	1038	8.7	11,899
≥4,000,000	3093	91.6	282	8.4	3375
Individual-level social capital					
Civic participation					
Yes	14,257	93.2	1037	6.8	15,294
No	13,444	87.9	1849	12.1	15,293
Social cohesion					
Yes	24,820	90.59	2578	9.4	27,398
No	2881	90.34	308	9.7	3189
Reciprocity					
Yes	27,382	90.6	2852	9.4	30,234
No	319	90.4	34	9.6	353
Presence of illnesses					
Yes	18,914	90.1	2085	9.9	20,999
No	7114	92.0	621	8.0	7735
Depression symptoms (GDS-15)					
Depression symptom (>5)	4676	87.8	649	12.2	5325
No depression	22,862	91.1	2223	8.9	25,085
Body mass index (kg/m^2^)					
<18.5	1538	89.99	171	10.01	1709
18.5–24.9	19,593	91.11	1912	8.89	21,505
≥25.0	5884	89.61	682	10.39	6566
Smoking habit					
Yes	2560	86.8	390	13.2	2950
No	24,308	91.0	2399	9.0	26,707
Alcohol consumption					
Yes	10,553	89.8	1199	10.2	11,752
No	16,928	91.1	1665	9.0	18,593
Daily walking time (min/day)					
<30	7229	87.9	1000	12.2	21,898
≥30	20,066	91.6	1832	8.4	8229
Frequency of going outside					
1 time or more per week	26,719	90.9	2673	9.1	29,392
Less than 1 time per week	826	81.3	190	18.7	1016

IADL: instrumental activities of daily living. GDS: Geriatric Depression Scale.

**Table 2:** Factor loadings of community-level social capital scale.

Variables	Exploratory Factor Analysis *		
	Social Cohesion	Civic Participation	Reciprocity
Community trust	0.820	0.101	0.076
Norms of reciprocity	0.851	−0.029	−0.076
Community attachment	0.765	−0.025	−0.021
Sports group	−0.026	0.781	−0.020
Volunteer group	0.060	0.398	0.047
Hobby activity	−0.001	0.887	−0.006
Received emotional support	−0.027	−0.082	0.883
Provided emotional support	−0.069	0.145	0.716
Receiver instrumental support	0.283	−0.050	0.425
Correlation coefficient			
Social cohesion & Civic participation	0.069 (*p* = 0.178)		
Social cohesion & Reciprocity	0.483 (*p* < 0.001)		
Civic participation & Reciprocity	0.278 (*p* < 0.001)		

* Exploratory factor analysis; Promax rotations and maximum likelihood method were applied.

**Table 3:** Characteristics of community areas.

Variables	*N*	Mean	(SD)	(Min–Max)
Community-level social capital				
Social cohesion (factor score)	380	−0.0021	(0.93)	(−2.67–2.61)
Civic participation (factor score)	380	−0.0027	(0.92)	(−2.97–3.87)
Reciprocity (factor score)	380	−0.0014	(0.91)	(−3.79–2.05)
Average annual household income (1,000,000 yen)	380	2.55	(0.41)	(1.46–4.27)
Population density (persons per km^2^ of inhabitable area)				
<2040	96			
2040–6852	95			
6853–10,122	95			
≥10,123	94			
Area (km^2^)				
<1.256	96			
1.256–2.2563	94			
2.2564–5.134	95			
≥5.135	95			

SD: Standard deviation.

**Table 4:** Association of IADL disability with community- and individual-level variables determined by multilevel logistic-regression ^a^.

	Variables		Model 1			Model 2			Model 3	
			OR	(95% CI)		OR	(95% CI)		OR	(95% CI)
**Community-level independent variable**										
	Social cohesion		1.15	(1.07–1.24)		1.02	(0.94–1.10)		1.01	(0.93–1.09)
	Civic participation		0.81	(0.76–0.85)		0.89	(0.83–0.95)		0.90	(0.84–0.96)
	Reciprocity		1.10	(1.00–1.19)		1.07	(0.98–1.16)		1.07	(0.98–1.16)
**Community-level covariates**										
Average annual household income (1000 yen)						1.16	(0.99–1.35)		1.19	(1.02–1.39)
Population density (people/km^2^ of inhabitable area) (ref. ≥10,123)										
	<2040					1.82	(1.43–2.33)		1.77	(1.39–2.25)
	2040–6852					1.54	(1.25–1.90)		1.50	(1.21–1.84)
	6853–10,122					1.04	(0.83–1.29)		1.03	(0.82–1.28)
Area (km^2^) (ref. ≥5.135)										
	<1.256					1.05	(0.83–1.34)		1.04	(0.82–1.32)
	1.256–2.2563					1.00	(0.82–1.22)		1.00	(0.82–1.21)
	2.2564–5.134					1.09	(0.97–1.23)		1.10	(0.97–1.24)
**Individual-level independent variable (ref. no)**										
Social cohesion						0.87	(0.76–0.99)		0.95	(0.83–1.08)
Civic participation						0.64	(0.58–0.69)		0.68	(0.63–0.74)
Reciprocity						1.07	(0.73–1.56)		1.17	(0.80–1.71)
**Individual-level covariates**										
Sex (ref. women)										
	Men					2.45	(2.25–2.68)		2.55	(2.31–2.82)
Age (ref. 65–69)										
	70–74					1.24	(1.12–1.38)		1.22	(1.10–1.35)
	75–79					1.71	(1.53–1.91)		1.64	(1.46–1.83)
	80–84					3.00	(2.62–3.43)		2.83	(2.47–3.25)
	≥85					5.52	(4.47–6.82)		5.22	(4.21–6.48)
Marital status (ref. divorced)										
	Married					2.61	(1.85–3.67)		2.74	(1.94–3.87)
	Widowed					2.32	(1.63–3.31)		2.37	(1.66–3.38)
	Never married					1.36	(0.82–2.24)		1.36	(0.82–2.25)
Educational attainment (ref. 10–12 years)										
	<10					1.41	(1.29–1.55)		1.39	(1.27–1.53)
	≥13					0.92	(0.82–1.03)		0.92	(0.82–1.04)
Annual household income (yen) (ref. ≥4,000,000)										
	<2,000,000					1.09	(0.94–1.26)		1.04	(0.90–1.20)
	2,000,000–3,999,999					1.01	(0.87–1.16)		1.00	(0.87–1.15)
Presence of illnesses (ref. no)										
	Yes								1.13	(1.02–1.24)
Depression symptoms (ref. no depression) (GDS-5)										
	Depression symptom (GDS >5)								1.25	(1.13–1.38)
Body mass index (ref. 18.5–24.9)										
	<18.5								1.12	(0.94–1.33)
	≥25.0								1.15	(1.04–1.26)
Smoking habit (ref. no)										
	Yes								1.19	(1.06–1.35)
Alcohol consumption (ref. no)										
	Yes								0.91	(0.84–1.00)
Daily walking time (ref. ≥30 min/day)										
	<30								1.34	(1.23–1.46)
Frequency of going outside (ref. 1 time or more per week)										
	Less than 1 time per week								1.61	(1.35–1.92)
Random-effects parameters										
Community-level variance (standard error)			0.150	0.035		0.084	0.048		0.073	0.056

OR: Odds ratios; CI: Confidence interval; ref.: reference. a Random-effects of estimate (standard error) of Null model was 0.26 (0.035); Model 1: Each community-level social capital variable was incorporated into the model without adjustment; Model 2: Model 1 + community-level average annual household income, population density, area, individual-level social capital, sex, age, marital status, educational attainment, and annual household income; Model 3: Model 2 + presence of illnesses, depression symptoms, body mass index, smoking habit, alcohol consumption, daily walking time, and frequency of going outside.

## Supplementary Materials

The following are available online at https://www.mdpi.com/1660-4601/16/5/828/s1, Table S1: Sensitivity analysis: Association of IADL disability with community- and individual-level variables determined by multilevel logistic regression., Table S2: Subgroup analysis: Association between community- and individual-level variables and IADL disability by sex and age.
