# Supplementary material for: Does Community-Level Social Capital Predict Decline in Instrumental Activities of Daily Living? A JAGES Prospective Cohort Study
Source: Int J Environ Res Public Health. 2019 Mar 7;16(5):828. doi: 10.3390/ijerph16050828 (PMC6427449; doi:10.3390/ijerph16050828)
Supplement: Supplementary file 1 [file ijerph-16-00828-s001.pdf]

Table 1. Sensitivity analysis: Association of IADL disability with community- and individual-level variables determined by multilevel logistic regression [a].

Variables	Model 1		Model 2		Model 3	
	OR	(95% CI)	OR	(95% CI)	OR	(95% CI)
Community-level independent variable						
Social cohesion (ref. < −0.540)						
−0.540–0.115	1.29	(1.09–1.54)	1.05	(0.87–1.26)	1.04	(0.86–1.25)
0.116–0.6022	1.48	(1.24–1.76)	1.12	(0.93–1.35)	1.10	(0.91–1.33)
≥0.6023	1.55	(1.30–1.85)	1.10	(0.90–1.33)	1.07	(0.88–1.31)
Civic participation (ref. < −0.526)						
−0.526–0.012	0.81	(0.72–0.91)	0.86	(0.76–0.97)	0.86	(0.76–0.98)
0.0121–0.564	0.77	(0.68–0.87)	0.87	(0.76–0.99)	0.87	(0.76–0.99)
≥0.565	0.56	(0.48–0.64)	0.73	(0.61–0.87)	0.74	(0.62–0.88)
Reciprocity (ref. <−0.418)						
−0.418–0.190	1.20	(1.03–1.41)	1.13	(0.96–1.31)	1.11	(0.95–1.30)
0.191–0.559	1.18	(1.01–1.38)	1.05	(0.89–1.23)	1.04	(0.89–1.22)
≥0.560	1.20	(1.02–1.42)	1.12	(0.95–1.32)	1.12	(0.94–1.32)
Community-level covariates						
Average annual household income (1000 yen)			1.18	(1.01–1.37)	1.21	(1.05–1.41)
Population density (people/km^2 of inhabitable area) (ref ≥10,123)						
<2040			1.84	(1.45–2.35)	1.78	(1.40–2.27)
2040–6852			1.55	(1.25–1.91)	1.50	(1.22–1.85)
6853–10,122			1.05	(0.84–1.30)	1.04	(0.83–1.29)
Area (km^2) (ref ≥5.135)						
<1.256			1.09	(0.85–1.39)	1.07	(0.83–1.36)
1.256–2.2563			1.02	(0.83–1.24)	1.01	(0.83–1.23)
2.2564–5.134			1.09	(0.96–1.24)	1.10	(0.97–1.24)
Individual-level independent variable (ref. no)						
Social cohesion			0.87	(0.76–0.99)	0.94	(0.83–1.08)
Civic participation			0.63	(0.58–0.69)	0.68	(0.63–0.74)
Reciprocity			1.08	(0.74–1.58)	1.19	(0.81–1.73)
Individual-level covariates						
Sex (ref. women)						
Men			2.45	(2.24–2.68)	2.55	(2.31–2.82)
Age (ref. 65–69)						
70–74			1.24	(1.12–1.38)	1.22	(1.10–1.36)

75–79			1.71	(1.53–1.91)	1.64	(1.46–1.84)
80–84			3.00	(2.62–3.43)	2.83	(2.46–3.24)
≥85			5.52	(4.46–6.82)	5.22	(4.21–6.47)
Marital status（ref divorced）						
Married			2.60	(1.84–3.67)	2.73	(1.94–3.86)
Widowed			2.32	(1.63–3.30)	2.36	(1.65–3.37)
Never married			1.36	(0.82–2.25)	1.36	(0.83–2.26)
Educational attainment (ref. 10–12 years)						
<10			1.41	(1.29–1.55)	1.39	(1.27–1.53)
≥13			0.92	(0.82–1.03)	0.92	(0.82–1.04)
Annual household income (yen) (ref. ≥4,000,000)						
<2,000,000			1.09	(0.94–1.26)	1.04	(0.90–1.20)
2,000,000–3,999,999			1.01	(0.87–1.16)	1.00	(0.86–1.15)
Presence of illnesses (ref. no)						
Yes					1.13	(1.02–1.24)
Depression symptoms (ref. no depression) (GDS-5)						
Depression symptom (GDS >5)					1.25	(1.13–1.38)
Body mass index (ref. 18.5–24.9)						
<18.5					1.12	(0.94–1.33)
≥25.0					1.15	(1.04–1.26)
Smoking habit (ref. no)						
Yes					1.19	(1.06–1.35)
Alcohol consumption (ref. no)						
Yes					0.91	(0.83–1.00)
Daily walking time (ref. ≥30 min/day)						
<30					1.33	(1.22–1.45)
Frequency of going outside (ref. 1 time or more per week)						
Less than 1 time per week					1.61	(1.36–1.92)
Random-effects parameters						
Community-level variance (standard error)	0.117	0.039	0.058	0.067	0.043	0.091

OR: Odds ratios; CI: Confidence interval; ref.: reference. [a] Random-effects of estimate (standard error) of Null model was 0.26 (0.035). Model 1: Each community-level social capital variable was incorporated into the model without adjustment. Model 2: Model 1 + community-level average annual household income, population density, area, individual-level social capital, sex, age, marital status, educational attainment, and annual household income. Model 3: Model 2 + presence of illnesses, depression symptoms, body mass index, smoking habit, alcohol consumption, daily walking time, and frequency of going outside.

Table 2. Subgroup analysis: Association between community- and individual-level variables and IADL disability by sex and age.

Variables	Men [a] (*n* = 13,919)		Women [a] (*n* = 16,668)		Age 65–74 [b] (*n* = 22,180)		Age ≥ 75 [b] (*n* = 8407)	
	**OR**	**(95% CI)**	**OR**	**(95% CI)**	**OR**	**(95% CI)**	**OR**	**(95% CI)**
Community-level independent variable								
Social cohesion	1.05	(0.96–1.15)	0.92	(0.81–1.05)	1.01	(0.92–1.11)	1.01	(0.90–1.14)
Civic participation	0.93	(0.86–1.01)	0.83	(0.74–0.93)	0.90	(0.83–0.98)	0.90	(0.81–1.00)
Reciprocity	1.03	(0.93–1.15)	1.11	(0.96–1.28)	1.08	(0.97–1.20)	1.05	(0.93–1.19)
Community-level covariates								
Average annual household income (1000yen)	1.11	(0.93–1.34)	1.40	(1.09–1.80)	1.26	(1.04–1.54)	1.09	(0.87–1.37)
Population density (people/km^2 of inhabitable area) (ref. ≥10,123)								
<2040	1.54	(1.16–2.05)	2.54	(1.63–3.95)	1.46	(1.07–2.00)	1.98	(1.37–2.85)
2040–6852	1.24	(0.97–1.58)	2.32	(1.57–3.43)	1.24	(0.95–1.63)	1.62	(1.18–2.22)
6853–10,122	1.09	(0.85–1.40)	0.82	(0.52–1.30)	0.90	(0.68–1.21)	1.17	(0.84–1.64)
Area (km^2) (ref. ≥5.135)								
≤1.256	1.16	(0.87–1.54)	0.85	(0.54–1.33)	1.04	(0.77–1.42)	0.94	(0.65–1.37)
1.256–2.2563	1.06	(0.84–1.33)	0.94	(0.67–1.32)	1.12	(0.88–1.41)	0.84	(0.61–1.14)
2.2564–5.134	1.19	(1.02–1.37)	0.99	(0.81–1.21)	1.07	(0.92–1.24)	1.14	(0.94–1.38)
Individual-level independent variable (ref. no)								
Social cohesion	0.89	(0.75–1.06)	1.01	(0.81–1.26)	0.91	(0.77–1.07)	1.00	(0.79–1.26)
Civic participation	0.80	(0.72–0.89)	0.54	(0.47–0.63)	0.74	(0.66–0.83)	0.59	(0.51–0.68)
Reciprocity	0.81	(0.52–1.25)	1.71	(0.75–3.87)	0.97	(0.60–1.55)	1.49	(0.79–2.79)
Individual-level covariates								
Sex (ref. women)								
Men					3.94	(3.45–4.49)	1.55	(1.33–1.81)
Age (ref. 65–69)								
70–74	1.08	(0.95–1.22)	1.63	(1.35–1.98)				
75–79	1.21	(1.05–1.40)	3.01	(2.46–3.68)				
80–84	1.75	(1.46–2.09)	6.85	(5.45–8.62)				
≥85	2.40	(1.77–3.25)	16.06	(11.63–22.16)				
Marital status (ref. divorced)								
Married	4.46	(2.61–7.62)	1.74	(1.10–2.75)	3.07	(2.01–4.69)	2.63	(1.44–4.81)
Widowed	1.86	(1.05–3.31)	1.81	(1.14–2.88)	2.05	(1.30–3.23)	2.73	(1.49–5.00)
Never married	1.51	(0.71–3.31)	1.07	(0.54–2.13)	1.45	(0.79–2.69)	1.14	(0.47–2.75)
Educational attainment (ref. 10–12 years)								

<10	1.33	(1.18–1.50)	1.59	(1.36–1.85)	1.51	(1.34–1.70)	1.37	(1.18–1.60)
≥13	0.94	(0.82–1.08)	0.78	(0.59–1.02)	0.92	(0.79–1.07)	0.97	(0.79–1.18)
Annual household income (yen) (ref. ≥4,000,000)								
<2,000,000	1.10	(0.92–1.31)	0.98	(0.76–1.26)	1.24	(1.03–1.50)	0.80	(0.64–1.01)
2,000,000-3,999,999	1.06	(0.89–1.26)	0.95	(0.73–1.22)	1.13	(0.94–1.36)	0.84	(0.67–1.05)
Presence of illnesses (ref. no)								
Yes	1.10	(0.98–1.24)	1.16	(0.97–1.40)	1.14	(1.01–1.29)	1.13	(0.95–1.35)
Depression symptoms (ref. no depression) (GDS-5)								
Depression symptom (GDS >5)	1.19	(1.04–1.35)	1.47	(1.25–1.73)	1.19	(1.04–1.36)	1.35	(1.15–1.57)
Body mass index (ref. 18.5–24.9)								
<18.5	1.09	(0.85–1.39)	1.30	(1.01–1.68)	1.20	(0.94–1.54)	1.20	(0.94–1.53)
≥25.0	0.96	(0.85–1.08)	1.55	(1.32–1.82)	1.12	(0.99–1.26)	1.12	(0.95–1.31)
Smoking habit (ref. no)								
Yes	1.18	(1.04–1.34)	0.94	(0.59–1.50)	1.16	(1.00–1.33)	1.02	(0.81–1.30)
Alcohol consumption (ref. no)								
Yes	0.90	(0.81–1.00)	0.87	(0.71–1.08)	0.86	(0.77–0.97)	0.92	(0.79–1.07)
Daily walking time (ref. ≥30 min/day)								
<30	1.30	(1.17–1.45)	1.42	(1.23–1.63)	1.34	(1.19–1.50)	1.39	(1.22–1.58)
Frequency of going outside (ref. 1 time or more per week)								
Less than 1 time per week	1.52	(1.20–1.92)	1.63	(1.25–2.11)	1.51	(1.17–1.94)	1.68	(1.33–2.13)
Random-effects parameters								
Community-level variance (standard error)	0.023	0.225	3.85×10^{-7}	0.191	3.16×10^{-10}	0.104	0.062	0.129

OR: Odds ratios; CI: Confidence interval; ref.: reference. [a] Adjusted for each community-level social capital variable, community-level average annual household income, population density, area, individual-level social capital, age, marital status, educational attainment, annual household income, presence of illnesses, depression symptoms, body mass index, smoking habit, alcohol consumption, daily walking time, and frequency of going outside. [b] Adjusted for each community-level social capital variable, community-level average annual household income, population density, area, individual-level social capital, sex, marital status, educational attainment, annual household income, presence of illnesses, depression symptoms, body mass index, smoking habit, alcohol consumption, daily walking time, and frequency of going outside.
